# Influence of Regulated Deficit Irrigation and Environmental Conditions on Reproductive Response of Sweet Cherry Trees

**DOI:** 10.3390/plants9010094

**Published:** 2020-01-11

**Authors:** Victor Blanco, Pedro José Blaya-Ros, Roque Torres-Sánchez, Rafael Domingo

**Affiliations:** 1 Dpto Ingeniería Agronómica, Universidad Politécnica de Cartagena (UPCT), Paseo Alfonso XIII, 48, E-30203 Cartagena, Spain; pedro.blaya@upct.es (P.J.B.-R.); rafael.domingo@upct.es (R.D.); 2 Dpto Automática, Ingeniería Eléctrica y Tecnología Electrónica, Universidad Politécnica de Cartagena (UPCT), Campus de la Muralla s/n, E-30202 Cartagena, Spain; roque.torres@upct.es

**Keywords:** carbohydrates stock, chill hours, flowering, fruit setting, irrigation, leaf area, *Prunus avium* L.

## Abstract

The reproductive response of fifteen year old sweet cherry trees (*Prunus avium* L.) combination ‘Prime Giant’/SL64 under Mediterranean climate to deficit irrigation was studied in a commercial orchard in south-eastern Spain for four seasons. Three irrigation treatments were assayed: (i) control treatment, irrigated without restrictions at 110% of seasonal crop evapotranspiration; (ii) sustained deficit irrigation treatment, irrigated at 85% ET_c_ during pre-harvest and post-harvest periods, and at 100% ET_c_ during floral differentiation, and (iii) regulated deficit irrigation treatment, irrigated at 100% ET_c_ during pre-harvest and floral differentiation and at 55% ET_c_ during post-harvest. The duration and intensity of the phenological phases of sweet cherry trees, including cold accumulation, flowering, fruit set or fruit and vegetative growth, were assessed to ascertain whether the different irrigation strategies imposed affect the trees’ reproductive response (fruit yield, fruit size, leaf area, fruit physiological disturbances, and starch and soluble carbohydrates stock) in the same season or have a negative effect in the next season. Deficit irrigation did not advance, enhance or penalize flowering, fruit set or fruit growth. Neither did it diminish carbohydrate concentration in roots or cause an increase in the number of double fruits, which was more linked to high temperatures after harvest. However, deficit irrigation decreased vegetative growth and consequently the leaf area/fruit ratio, which, when it fell below 180 cm^2^ fruit^−1^, affected cherry size.

## 1. Introduction

In highly productive agricultural areas such as the Mediterranean Basin, where natural resources such as water are already scarce, climate change is a serious threat. Agroclimatic projection for the coming years foresees a significant increase in mean temperature, droughts, heat waves and a decrease in ground water availability in arid and semi-arid regions, which would include fruit-growing areas [1]. These changing conditions will modify expected rainfall patterns and increase extreme climatic events such as torrential rain, which, while contributing to an increase in accumulated annual precipitation, does not count as effective rain that contributes to the agronomical system, and, furthermore, the water cannot be collected [2]. Agricultural production and development are highly dependent on water availability, and, in a scenario of climate change vulnerability, irrigation strategies whose aim is to increase water use efficiency need to be adopted. In this regard, regulated deficit irrigation (RDI) is a promising irrigation strategy which has been seen to achieve water savings of up to 40% without decreasing fruit yield and to prevent excessive vegetative growth in numerous fruit trees and vines [3]. It may even enhance fruit quality at harvest [4] or during storage [5,6].

In 2017, Spain was the largest fresh fruit producer of all the European Union [7]. As regards sweet cherries, Spain is the fourth biggest producer worldwide, and although cultivation of this fruit is restricted to specific regions, new cultivars with lower chill requirements have led to this fruit tree being grown in warmer areas where it has not traditionally been cultivated, with the added advantage of an earlier bloom and harvest. In this respect, ‘Prime Giant’ is a promising sweet cherry cultivar of local importance in south-eastern Spain, which is highly appreciated by consumers and has previously been described as suited to warm winters [8]. These characteristics make this cultivar suitable for cultivation in semi-arid areas with hot dry summers and mild winters.

In these areas, where water is a limited resource, it is common for fruit growers to apply an uncontrolled water deficit during the postharvest period coinciding with summer. Furthermore, during these months very little or no rain usually falls and heat waves are quite common. However, if a water deficit is applied, it is important to know the effects on the same and the following year’s flower bud development, flowering, fruit set, fruit and leaf area growth and the possible presence of physiological disturbances. Indeed, extreme weather conditions and/or water deficits applied during the trees’ productive cycle might affect their response in the following year. In fruit crops such as pomegranate, mild deficit irrigation early in the growing season increases the number of flowers and fruit set but delays the first wave of blooming. However, severe deficit irrigation has been seen to result in a low number of flowers and heterogeneous fruit growth [9]. In loquat, deficit irrigation brings flowering forward without affecting fruit set [10]. In almond, an excessive water deficit during the previous summer affects flower development and decreases the following year’s fruit set and yield [11]. Similarly, in apricot, an extremely low soil water content during summer (flower bud formation) has been closely related to a high bud drop the following season [12]. Regulated deficit irrigation is also related to lower vegetative growth, and consequently with a lower leaf area. In sweet cherry this is important because decreases in the leaf area per fruit (LA: F) have been reported to negatively influence fruit size and quality [13]. It is therefore important to study the effect of deficit irrigation on leaf area, the relationship between leaf area and the number of fruit and to ascertain the threshold values in order to know the effects of a lower leaf area on fruit yield and quality.

Weather conditions are also extremely important. Thus, in stone fruit trees several physiological disturbances, such as flower bud drop, erratic bloom, floral anomalies and low fruit set, have been linked to mild winters and insufficient chilling which cannot overcome endodormancy [14,15]. In sweet cherry trees, warm winters and hot summers negatively affect flower bud development and final yield. In the cultivar ‘Marvin’, Alburquerque et al. [16] reported irregular flowering and yield with values under 1100 Chill Units and Beppu et al. [17] related the occurrence of double fruit with high temperatures during the previous postharvest. Moreover, Hedhly et al. [18] reported the effect of warm temperatures during bloom and how they drastically decreased fruit set.

The main aim of this work was to ascertain how deficit irrigation strategies affect the generative or reproductive response of ‘Prime Giant’ sweet cherry in semiarid zones. This research focuses on four different goals: (i) tree reproductive growth, (ii) fruit defects, (iii) roots starch concentration and its influence on the following yield, and (iv) the relationship between crop phenological responses and weather conditions during four consecutive seasons in the southeast of Spain.

## 2. Results

### 2.1. Water Applied and Meteorological Conditions

**Figure 1:**
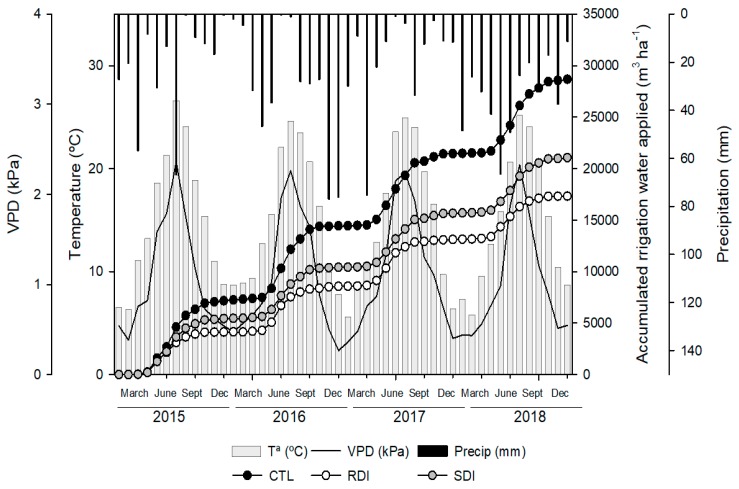
Seasonal evolution of monthly mean temperature (T^a^), monthly mean air vapour pressure deficit (VPD) accumulated monthly rainfall (Precip) and accumulated irrigation water applied to each treatment during the experiment (2015–2018) at the ‘Prime Giant’/SL64 sweet cherry orchard in Jumilla (Spain).

The average water applied by irrigation to each treatment and each season was 6890, 4152 and 5007 m^3^ ha^−1^ in the control (CTL), regulated deficit irrigation (RDI) and sustained deficit irrigation (SDI) treatments, respectively (Figure 1). The greatest difference in water applied among seasons to the same treatment was 491 m^3^ ha^−1^ to the trees irrigated under the SDI strategy, with the highest amount of water applied in 2017 and the lowest in 2018. The annual accumulated ET_0_ was 1271, 1247, 1248 and 1181 mm in 2015, 2016, 2017 and 2018, respectively. Accumulated precipitation was not sufficient to satisfy crop evapotranspiration and was the meteorological parameter that varied the most, with substantial inter-annual differences. For example, in 2017 accumulated rainfall was 264 mm, while in 2018 it was 405 mm.

Seasonal weather patterns were similar during the four years of study and typical of a semi-arid Mediterranean-type climate with dry and warm summers and mild winters (Figure 1). The daily mean temperature ranged from 1.1 °C in January to 29.1 °C in July. However, in summer, between July and August, maximum temperatures exceeded 35 °C. Similarly, VPD ranged from 0.06 to 4.04 kPa.

### 2.2. Chilling Requirements and Heat Accumulation until Full Bloom

According to the study location, the chilling accumulation period starts in November and finishes when 50% of the buds are at the green tip stage, in ‘Prime Giant’; this lasted throughout March. No effect of postharvest deficit irrigation bringing budbreak forward was observed. Thus, Table 1 shows the chilling hours, chilling units and chilling portions calculated each year of the study at the orchard. Among the three methods used to calculate chilling requirements, the highest CV was obtained by CH, and the lowest by the Dynamic model. The Dynamic model remained practically unchanged at 94 chill portions throughout the four years of the study, which corresponded to 1270 h below 7.2 °C and 1129 chill units.

Heat accumulation as growing degree hours (GDH) from the green tip stage until full bloom was calculated each year and varied between 6363 and 7014 GDH in 2017 and 2016, respectively. The full bloom date differed by 13 days between the year with the earliest full bloom and the year with the latest (Table 1). Despite the differences in bloom date, neither flowering and nor fruit bearing was affected by frost damage any year.

### 2.3. Flower Density, Number of Flower Buds per Spur and Fruit Setting

The different irrigation strategies assayed did not result in significant differences among irrigation strategies for any of the measured parameters in any year of the study. However, there were significant differences in fruit setting between years (Table 2). Parameters such as flower density were not influenced by irrigation treatment or year; however, in two out of the three years, the CTL treatment provided lower values of spur per linear meter than the other treatments. It was observed in control trees that this lower value was not due to a low number of spur but to higher vegetative growth, which increases the distance between spurs and, consequently, results in a lower density. SDI trees provided slightly higher mean values for flowers than trees of the other treatments (Table 2). There were no differences among years in the number of flower buds per spur or the number of flowers per bud, which suggests that these characters could be genotype-dependent. On the other hand, fruit set percentage was influenced by the year, the values being between 52% and 89% higher in 2016 than in previous and following years.

### 2.4. Fruit and Vegetative Growth

**Figure 2:**
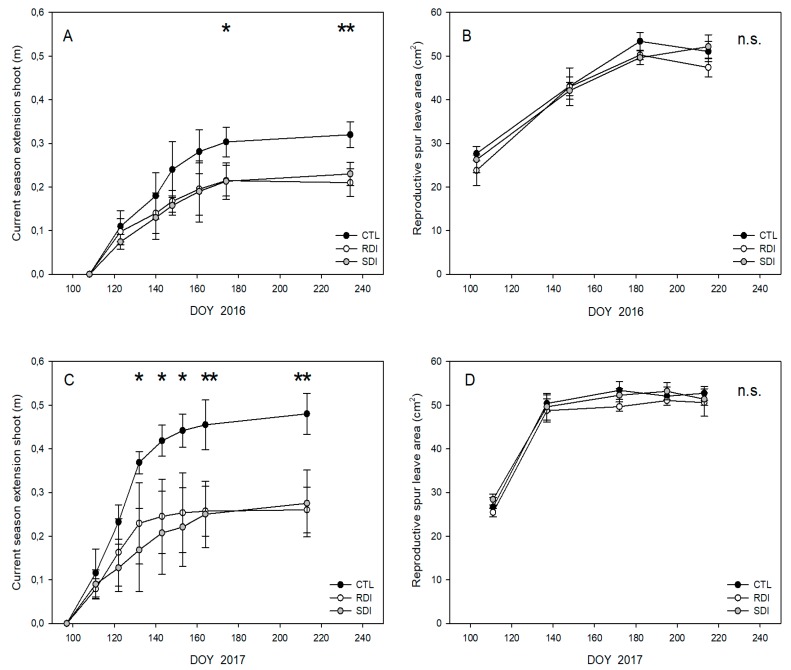
Seasonal evolution of accumulated length of current season shoots (**A**,**C**) and reproductive spur leaf area (**B**,**D**) of ‘Prime Giant’/SL64 sweet cherry combination for 2016 and 2017 seasons to CTL, RDI and SDI irrigation treatments. Each observation corresponds to an average value for each treatment (n = 4). * and ** refer to significant effects at each time according to ANOVA (*p* < 0.05 and 0.01, respectively). n.s. refers to not significant differences.

Trees of the CTL treatment gave longer shoots than those of the deficit treatments, particularly in 2017 when the shoots of CTL trees were 50% longer than those of SDI and RDI. There were no differences between SDI and RDI at harvest, but shoots of SDI trees showed slower growth during preharvest (Figure 2A,C). No differences in the leaf area of a reproductive spur were detected among treatments or years (Figure 2B,D), with mean values close to 50 cm^2^ per leaf once the final size was achieved.

Regarding fruit growth, the length of the fruit development stages was similar among fruits of different irrigation treatments. In 2017, the full bloom stage started 3, 14 and 9 days earlier than in 2015, 2016 and 2018, respectively. However, these differences in days were not maintained until harvest. In 2017, the harvest days were 1, 16 and 12 days earlier than in 2015, 2016 and 2018, respectively. Consequently, fruit development from the bloom stage to harvest lasted from 58 to 63 days, depending on the year (Table 3). Despite the differences in natural days, when growing degree days (GDD) were calculated all the years provided similar results. Consequently, it can be stated that ‘Prime Giant’ sweet cherry trees in our study required 779 ± 36 GDD to reach complete fruit development from bloom stage to harvest (Table 3).

**Figure 3:**
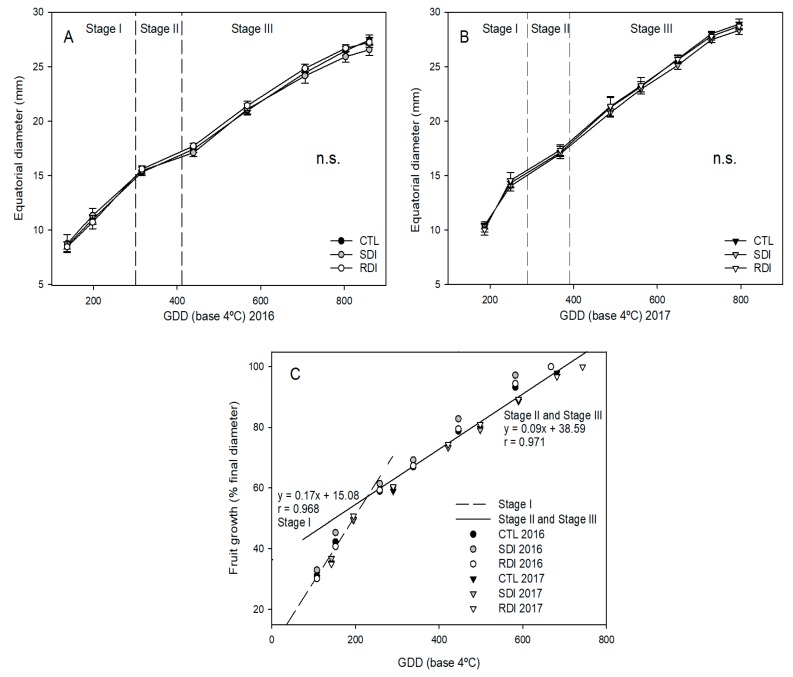
Seasonal fruit growth of ‘Prime Giant’/SL64 sweet cherry combination during 2016 (**A**) and 2017 (**B**) to CTL, SDI and RDI irrigation treatments and fruit growth pattern related to growing degree days (GDD) (**C**). Each observation corresponds to an average value for each treatment (n = 4). n.s. refers to not significant differences among irrigation treatments, according to ANOVA (*p* < 0.05).

The years 2016 and 2018 had slightly a higher GDD accumulation during fruit development than 2015 and 2017. In 2016 and 2018, fruit development took place in warmer days, particularly during Stage III of fruit development, from late May to mid-June. However, in 2015 and 2017, Stage III lasted from late April to late May, so GDD accumulation was lower and this stage lasted longer (Figure 3A,B). As expected, fruit growth was strongly related to GDD accumulation and two linear relationships were obtained, the first up to Stage I and the second to Stage II and Stage III of ‘Prime Giant’ sweet cherry development (Figure 3C).

### 2.5. Leaf Area and LA:F Ratio

**Figure 4:**
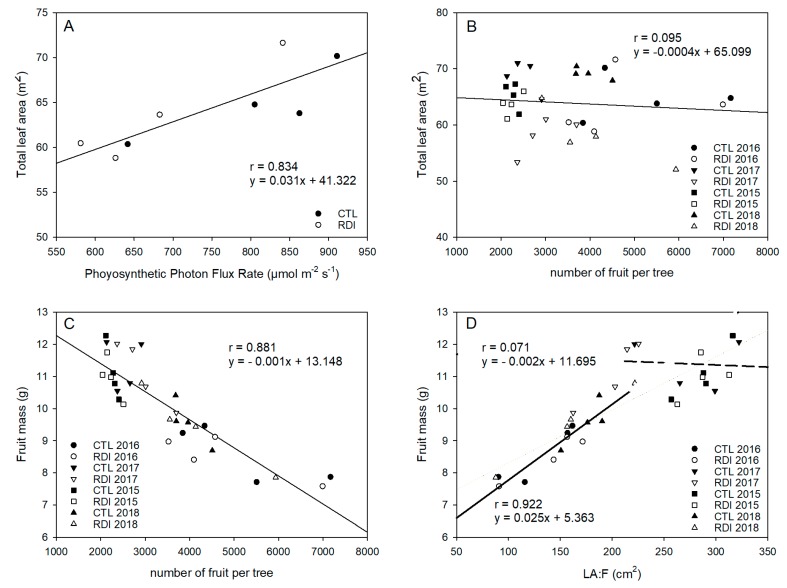
Relationships between photosynthetic photon flux rate and tree total leaf area (**A**), number of fruit per tree and total leaf area (**B**), number of fruit per tree and fruit unitary mass (**C**), leaf area:fruit ratio (LA:F) and fruit unitary mass (**D**). Each observation corresponds to an average value for each block (n = 3).

RDI and CTL resulted in similar total leaf areas in 2015 and 2016; however, in 2017 and 2018, CTL trees had a significantly higher leaf area and shaded area than RDI trees (Figure 4A). There were no differences among irrigation treatments in terms of yield in any year of the study. However, 2016 and 2018 were high cropping years, and the mean yield and mean number of fruits per tree were 56% and 94% higher in these years than in 2015 and 2017, which consequently modified the leaf area to fruit ratio (LA:F). Therefore, there was no relationship between total tree leaf area and number of fruit per tree (Figure 4B), but fruit mass was strongly influenced by the LA:F (Figure 4C). 

When the LA:F ratio was calculated, it was observed that, in 2016 and 2018, CTL and RDI trees resulted in mean LA:F values close to 130 cm^2^ fruit^−1^. On the other hand, in 2015 and 2017, as a result of the lower fruit load, trees of both treatments resulted in significantly higher LA:F ratios. Furthermore, the effect of deficit irrigation on RDI trees resulted in a lower total tree area and significantly lower values of LA:F than CTL trees, with 272 and 194 cm^2^ fruit^−1^ for CTL and RDI trees respectively. The LA:F ratio for both years was compared with fruit unitary (Figure 4D). Thus, two different situations could be distinguished, depending on fruit unitary mass. The first relationship showed a strong and positive linear relationship between LA:F and fruit mass, where higher LA:F values resulted in larger fruit, until it reached values close to 180 cm^2^ fruit^−1^, which corresponds to a fruit unitary mass of 10 g (optimal mass of ‘Prime Giant’ sweet cherries). Above this value, this relationship was not observed, and fruit size did not seem to be as influenced by leaf area. Consequently, further increases in leaf area per fruit above 180 cm^2^ fruit^−1^ did not result in higher fruit mass.

### 2.6. Double Fruits Incidence

**Figure 5:**
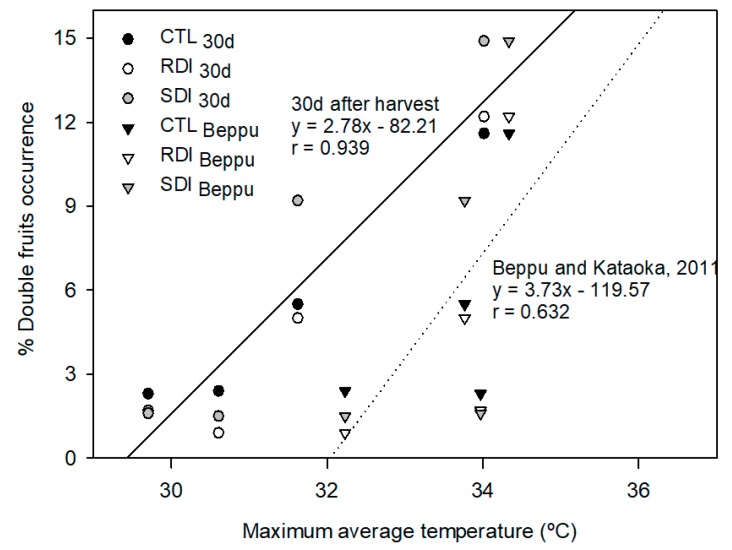
Relationships between maximum average temperature during the 30 days after harvest or 30 days between mid-July and mid-August (period reported by Beppu and Kataoka [19] and the % of double fruits occurrence. Each observation corresponds to the average value for each treatment and year (n = 4).

Double fruits were strongly related to the average maximum temperature in the previous early summer during a period of 30 days after harvest. This relationship weakened when other periods, such as the warmest days of the year, were considered (Figure 5). There were no differences in double fruits occurrence among trees of different irrigation treatments. Postharvest deficit irrigation did not result in a significantly higher percentage of double fruits in the total tree yield, which varied from 2% in 2017 to 12% in 2015. However, in 2018, SDI trees, which were the only trees submitted to slight deficit irrigation during preharvest, produced 66% more double fruit, 9%, than those produced by the trees from CTL and RDI (5% and 5.5% respectively).

### 2.7. Starch and Soluble Carbohydrates Concentration (SSCC)

Irrigation treatment did not result in significant differences in root carbohydrate concentration in any year of the study. However, there were differences between years. In 2016 and 2018, the carbohydrate concentration in roots was significantly higher than in 2017. The high SSCC measured in the winters of 2016 and 2018, resulted in a high fruit setting and fruit load the subsequent season. On the other hand, as a consequence of the high fruit load measured in 2016, it was possible to say that SSCC decreases after a high cropping year, and in 2017 the SSCC was significantly lower than that measured the previous and following years.

Fruit set and fruit load varied significantly throughout the experiment. They were higher in 2016 and 2018 than in 2015 and 2017, which matches the years with higher SSCC values. In 2015 and 2017, mean fruit load was 2513 ± 138 fruits per tree. However, in 2016 and 2018 it was 4867 ± 187 fruits per tree. CTL resulted in higher mean values of SSCC, but no significant differences resulted from the irrigation treatments. 

**Figure 6:**
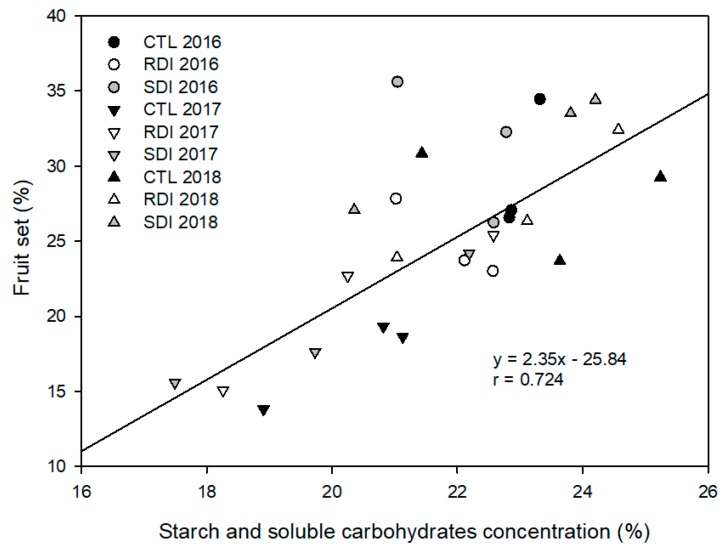
Relationship between starch and soluble carbohydrates concentration (%) in winter (January) and fruit setting in spring of the same year. Each observation corresponds to an average value for each block (n = 2).

Although fruit set and fruit load were not influenced by the irrigation strategy applied, they were strongly influenced by the winter SSCC, and a linear relationship was found between winter SSCC and fruit setting the subsequent season (Figure 6). Thus, in 2017, when fruit set was a third lower than in 2016 and 2018, SSCC was lower than 20%, while in the other two years SSCC mean values were higher.

## 3. Discussion

Despite slight differences among years, climatic conditions were similar as regards both dry springs and summers with high evaporative demand and VPD (Figure 1), and mild winters. When climatic data were analyzed and all the chill methods were compared, the Dynamic model was the method that showed the lowest coefficient of variation (CV). The Dynamic model and the chill portions have previously been reported as the being best method and unit to measure chilling requirements in warm areas [20]. Although the low statistical error (SE) obtained suggested it was a useful model for predicting the bloom date, this model is not yet widely used by producers. In this sense, when we compared the results between the chill hours model and the chill units model, our results differed from those of Alburquerque et al. [16], who found that the chill hours model was not suitable for calculating sweet cherry chill requirements. Regarding the climatic data used, the three methods showed low values of CV, and there were no differences between the chill hours and the chill units models (Table 1). Heat requirements were also similar among years. The cultivar used in this work, ‘Prime Giant’, needed a lower accumulation of GDH between the end of dormancy and full bloom than those values reported for cultivars such as ‘Burlat’ and ‘Marvin’, with similar blooming dates, in early April [16]. The rapid development of ‘Prime Giant’ sweet cherries during the stage from swollen bud to open flower makes this cultivar vulnerable to frost injury as it can complete its heat requirements easier than other cultivars during a period of warm days in winter (false spring), and therefore blooms early. Frequently, early blooming in sweet cherry trees is followed by a damaging freeze as the tree responds to warmer temperatures with budbreak and then faces the risk of the temperature dropping [21]. This characteristic limits ‘Prime Giant’ sweet cherry cultivation in cold areas where frosts and air temperatures below zero are common during early spring.

Bloom was not significantly affected by the irrigation treatments imposed in any year of study. Moreover, it lasted between seven and eight days in all treatments (Table 3), meaning that bloom duration was not influenced by irrigation. Regarding flower density, RDI has been reported as an irrigation strategy that enhances flowering in fruit trees such as pomegranate [9], loquat [10], pear [22], mango [23] and apricot cultivars such as ‘Sundrop’ [24]. However, in other fruit trees such as plum, or other apricot cultivars such as ‘Guillermo’, deficit irrigation during postharvest has been seen to have no effect on bloom and flower density and, even when water stress was excessive, the flowering date was delayed and flower density diminished [12,25]. In our experiment, deficit irrigation had no effect on sweet cherry flower density, number of flowers or fruit set. Although there were no significant differences among treatments, CTL trees showed the lowest results and trees submitted to SDI had a 16% higher number of spurs, 5% more flowers per bud and 15% higher number of fruits per flower compared to CTL (Table 2). The lower number of spurs per linear meter of branch in CTL trees could be due to the higher vegetative growth measured in these trees. On the other hand, the greater number of flower buds and flowers per tree in SDI trees could be a slight response to preharvest deficit irrigation in the following year’s flowering. In sweet cherry, flower induction and differentiation are faster than in other fruit crops [26]. Flower induction starts the previous year while the fruit is developing on the tree and finishes at early postharvest; it has been reported that, at harvest, most of the buds have already formed flower primordia [27,28]. Consequently, we hypothesize that a slight water deficit applied during the late preharvest in SDI trees could have an effect on flower induction and consequently increase the number of fruits per tree. We can exclude the idea that the increase might be explained as a consequence of deficit irrigation during flower differentiation in postharvest, as this was not observed in RDI trees, which were submitted to a stronger postharvest water deficit than SDI trees.

Similarly to full bloom date, it should be noted that harvest date also varied among years, by up to 16 days if we compare the earliest and latest harvest dates, depending on weather conditions, which, in a rapidly developing fruit, represents a great variation. On the other hand, when GDD accumulation was compared between years, it variated by less than 8% (Table 3). As expected, and has been reported for other stone fruit such as apricot and peach [6,29], the use of methods that involve the accumulation of energy units to predict fruit growth evolution, rather than using dates, results in a smaller variation. Consequently, when fruit growth was seen alongside GDD accumulation two equations could be distinguished depending on the fruit growth pattern. The first one refers to Stage I, the exponential fruit growth caused by cell division, which showed the greatest slope. The second equation refers to Stage II and Stage III, pit hardening and cell enlargement (Figure 3C). There were no significant differences in fruit development or in fruit size among irrigation treatments. However, particularly in high cropping years such as 2016, the fruit of SDI trees showed a trend towards a lower mean unitary size than the fruit of CTL and RDI trees (Figure 3A). 

Current season shoot growth and leaf expansion have been reported to be sensitive indicators of water stress in most temperate fruit trees, and their reduction is one of the first physiological responses of trees to water deficit, even before stomatal closure and photosynthesis inhibition [30]. In this sense, the assayed irrigation strategies affected vegetative growth, particularly current season shoot growth (Figure 2), which has been reported as a sensitive indicator of water stress in sweet cherry trees [31]. Thus, RDI resulted in a lower leaf area than was seen in CTL trees (Figure 4A). RDI strategies have been reported as an effective method to control excessive vegetative growth and vigor in sweet cherry trees [32,33,34]. However, in sweet cherry, fruit size is strongly linked to LA:F ratio, so an excessive reduction in the total leaf area could result in the production of small fruit, particularly in high cropping seasons when the LA:F ratio is low and the competition for photoassimilates among fruits would generate a source-sink limitation [35,36]. Our results show that LA:F ratios up to 180 cm^2^ fruit^−1^ for ‘Prime Giant’/‘SL64’ did not limit fruit unitary mass and ensure the availability of photoassimilates to obtain high quality fruit (Figure 4D). Higher LA:F values, such as those reported for the combinations ‘Bing’/‘Gisela 5’ and ‘Royal Dawn’/‘MaxMa14’ [13,37] did not increase fruit size. On the other hand, lower LA:F values as in the combination ‘Lapins’/‘Gisela 5’ of 99 cm^2^ fruit^−1^ [38] strongly penalized fruit size, and gave a fruit unitary mass lower than 8 g.

Considering all the data, the lower mean unitary weight of the cherries of SDI trees might be explained by the cumulative effect of several factors influenced by the deficit irrigation strategy applied. We have already related that SDI led to the trees to a slightly greater number of flowers per tree and corresponding fruit setting (Table 2) which resulted in a greater number of fruits per tree, but also decreased vegetative growth (Figure 2), and consequently the sink/source balance could have been disrupted. Small differences in equatorial diameter, although not statistically significant, should be kept in mind, as sweet cherry profitability is strongly related to fruit size [39].

Another factor that influences sweet cherry profitability is the occurrence of physiopathies, such as double fruit occurrence. In peaches, double fruiting has been related to water stress during postharvest [40]. However, in nectarine postharvest deficit irrigation did not increase the number of double fruits [41]. Similarly, in sweet cherry, we did not notice any significant effect of the irrigation treatments imposed on double fruit. Air temperature was the main factor affecting double pistil formation, which agrees with the results reported by Beppu and Kataoka [19], who stated that air temperatures above 30 °C during flower bud differentiation affect pistil formation.

The same authors claimed that the days when high temperatures are critical are from mid-July to mid-August of the previous year, and the higher the temperature during those days, the higher the frequency of double fruiting. We found that the period mentioned by Beppu and Kataoka [19] did not apply to the cultivar ‘Prime Giant’. However, when the maximum average temperature of the first 30 days after harvest was considered, the relationship obtained between double fruit occurrence and average maximum temperature was similar to that reported (Figure 5). As the harvest date varied by 16 days between the year with the earliest harvest (2017) and the latest harvest (2016), we consider double pistil development in ‘Prime Giant’ sweet cherry is more related to the temperature during the first 30 days of postharvest than the specific 30 days between mid-July and mid-August.

Apart from the frequency of double fruits, another serious problem in sweet cherry cultivation in warm areas is poor fruit set, which in semi-arid areas is more unstable and fluctuates widely between years [19]. Our results agree with that statement, as fruit setting was significantly different between years but not among irrigation treatments in any one year of the study (Table 2). In this sense, air temperature during the period from bud burst to petal fall has been mentioned as a factor to consider in fruit setting; however, we emphasize the influence of SSCC in fruit setting, as a relationship between fruit setting and SSCC was evident. Thus, we observed that, regardless of the irrigation treatment evaluated after a high cropping year, the tree cannot replenish the carbohydrate reserves consumed which consequently affects the following year’s fruit setting. The linear relation obtained between winter SSCC and the subsequent fruit set is similar to that reported by Marsal et al. [42] for the combination ‘Summit’/‘SL64′. In that work, fruit set was only related to starch concentration, while in our relation both starch and soluble carbohydrates are considered. It is well known that fruit set, as well as flowering and first vegetative development, mainly depend on the carbohydrate reserves, so we hypothesized that deficit irrigation could affect SSCC concentration in roots. The fruit act as a sink, and the only available source at those early stages are the SSCC stored during the previous season. Contrary to the results reported by Marsal et al. [42], in our experiment postharvest deficit irrigation did not penalize SSCC. However, our results matched those reported for peach under postharvest deficit irrigation [43]. 

## 4. Materials and Methods 

### 4.1. Experimental Site and Plant Material 

The experiment was conducted during four growing seasons (2015–2018) in a fifteen year old commercial orchard located in Jumilla (Murcia, Spain), latitude 38° 8′ N, longitude 1° 22ʹ W and 675 m above sea level, with a typical Mediterranean climate. Soil and irrigation water were characterized. Soil texture was sandy-loam (67.5% sand, 17.5% silt and 15% clay), and moderately stony. Low salinity irrigation water (electrical conductivity of 0.8 dS m^−1^) with concentrations of sodium and chloride below 1.7 and 1.05 mmol L^−1^, respectively, was used. 

The plant material consisted of sweet cherry trees (*Prunus avium* L.) of the combination ‘Prime Giant’/SL64 trained to a vase system with ‘Early Lory’ and ‘Brooks’ as pollinizers at a tree density of 667 trees ha^−1^.

Trees were automatically drip irrigated using a single drip line with three pressure-compensated emitters of 4 L h^−1^ each per tree. The different irrigation treatments were initiated each season before flowering and suspended at the end of November. Trees of all irrigation treatments were similarly managed by the technical department of the commercial orchard according to standard fruit farming practices for the cultivar and region. Fertilization, which consisted of 63, 30, 107 and 8 kg ha^−1^ of N, P_2_O_5_, K_2_O and CaO respectively, was applied through the irrigation system with the water and was the same in all treatments regardless of the amount of water applied.

### 4.2. Irrigation Treatments

The crop evapotranspiration (ET_c_) was calculated as the product of the reference evapotranspiration (ET_0_), the crop-specific coefficient (K_c_)—which, in the case of sweet cherry trees was reported by Marsal [44]—and a localization factor related to the percentage of ground covered by the crop, as reported by Fereres et al. [45].

The experiment consisted of three irrigation regimes: a control (CTL), irrigated at 110% ET_c_ to maintain non-limiting soil water conditions throughout the season; a regulated deficit irrigation treatment (RDI), which applied 100% of ET_c_ during preharvest and flower differentiation and 55% of ET_c_ during postharvest; and a sustained deficit irrigation treatment (SDI), which irrigated at 85% of ET_c_ during preharvest and postharvest, except for the 15–20 days after the first harvest (floral differentiation), when trees were irrigated at 100% ET_c_.

Treatments were distributed according to a completely randomized block design with four blocks per treatment. Each block consisted of seven trees, but only the five central trees were used for experimental measurements. 

### 4.3. Weather Conditions

Daily agrometeorological data such as temperature, relative humidity, rainfall, wind speed, radiation and ET_0_ were recorded by a weather station near the experimental orchard owned by the Spanish Consultancy Service for Irrigation Users (SIAR; http://crea.uclm.es/siar/datmeteo/).

The quantification of chill requirements was calculated by three different methods: (i) the chilling hours model described by Weinberger [46], as the number of hours below 7 °C and above 0 °C; (ii) the modified chill unit model [47], which considers positive and negative chill unit contributions depending on air temperature during the dormancy period; and (iii) the chill portions model [48,49] calculated as a dynamic model that considers two stages, a first, reversible, stage in which high air temperature destroys chill accumulation, and a second, irreversible, stage. Heat requirements were calculated as the accumulation of GDH based on average hourly air temperature minus 4 °C. The accumulation of GDH was considered from the end of dormancy (buds at BBCH stage 53) to the date when 50% of the flowers were open. The phenological calendar was expressed as day of the year (DOY) and days after full bloom (DAFB).

### 4.4. Flowering and Fruiting 

Two principal branches, randomly selected from east and west exposures in three trees per block, were used to measure the total number of fruiting spurs, flower buds per fruiting spur, flowers per flower bud and total number of fruits finally developed. The fruit set was calculated as the final number of fruit developed on the fruiting spurs on the branch divided by the total number of flowers counted, multiplied by one hundred.

### 4.5. Fruit and Vegetative Growth

Fruit growth was evaluated weekly during 2016 and 2017 preharvest as fruit unitary weight. Weekly, ten representative sweet cherries per block, forty per treatment, were picked and weighed on an electronic balance (AX623, Sartorius AG, Gottingen, Germany). 

Vegetative growth was evaluated by measuring the evolution of current season shoot length, reproductive spur leaf area and total leaf area. Current season shoot growth was periodically measured during the growing seasons of 2016 and 2017 in the same four shoots per tree, two trees per block, with a tape measure (Tylon Pocket, Stanley, New Britain, CT, USA). Similarly, in 2016 and 2017, 25 spur leaves per block were periodically cut off in order to be later scanned in the laboratory using a leaf-area meter (LI-3100C, LI-COR Inc., Lincoln, NE, USA). In the last measurements, when mature leaves showed a constant area, the fresh and dry weights of the same samples were measured to obtain a relationship between leaf area and fresh leaf weight and leaf area and dry leaf weight. Fresh and dry leaves were weighed on an electronic balance (AX623, Sartorius AG, Gottingen, Germany). Dry leaf weight was measured once the leaves were dried to a constant weight in a ventilated oven (Digitheat, JP Selecta SA, Barcelona, Spain) at 60 °C.

In order to estimate the total leaf area of the trees, the shaded area of the trees was measured during the four years of the study in the same three trees per block on clear days at noon with a linear quantum sensor (Accupar Linear PAR, Decagon Devices Inc., Pullman, WA, USA), which measures the radiation that passes through the canopy. The photosynthetic photon flux rate (PPFR) intercepted by the canopy was calculated as the value measured when the sensor is exposed directly to sunlight (1500 μmol m^−2^ s^−1^) minus the mean value measured from 30 measurements in a grid pattern (0.25 m^2^ mesh) under the tree projection on the soil surface. In 2016, the same trees in which PPFR was measured were chosen to calculate their total leaf area. Thus, during the annual pruning, the leaves were separated from the pruning wood and weighed in the field in order to obtain the corresponding weight of the wood and the leaves. From the fresh weight of the leaves the leaf area was estimated. Moreover, during autumn and winter the trees were covered by a plastic bird net to ascertain the total number of fallen leaves of each tree. The fallen leaves were periodically collected and taken to the laboratory, where they were dried to a constant weight. Based on the dry weight of these leaves, the leaf area was estimated. The total leaf area of the tree was calculated as the sum of the areas of the leaves of the pruning wood and the fallen leaves. The value of total leaf area per tree was related to the PPFR measured in the same tree.

### 4.6. Yield

The total fruit yield was determined each year of the study on the basis of the weight of the total fruit harvested per tree, measured in the five central trees of each block. Fruit unitary weight at harvest was calculated by counting the number of fruits in 5 kg samples from the three central trees of each block. The number of double cherries in those sample was recorded to evaluate the effect of the irrigation strategy on double fruit occurrence. Fruit load, taken as the number of fruits per tree, was estimated by the ratio of total fruit yield per tree to average fruit unitary weight. 

### 4.7. Starch and Soluble Carbohydrates Concentration

In January 2016, 2017 and 2018, active roots from two trees of three blocks per treatment were removed in order to analyze the tree’s total carbohydrate content before bloom. Active roots with a mean diameter of 5 mm, located at 20 cm depth within the wet area of the closest emitter to the trunk, were excised. In the laboratory, they were washed and dried in a forced air oven at 65 °C until a constant mass was reached. Dried root samples were ground with a hand mill (M20, IKA-WERKE, Staufen, Germany). Root total carbohydrates were calculated as the sum of soluble carbohydrates and starch, which were determined according to the Somogyi–Nelson method [50].

### 4.8. Statistical Analysis

Analysis of variance (ANOVA) and post-hoc test (Duncan’s multiple range) with a significance level of *p* < 0.05 were performed using the statistical software package IBM SPSS Statistic 24 (IBM Corp., Armonk, NY, USA) and linear relationships between variables were calculated with Sigma Plot 12.5 (Systat software Inc., San Jose, CA, USA).

## 5. Conclusions

‘Prime Giant’ sweet cherry trees could be recommended in semiarid areas which satisfy 94 chill portions. However, as this cultivar rapidly develops from the swollen bud to open flower stage, it cannot be proposed as a suitable cultivar in areas with late frost episodes that may coincide with blooming. Moreover, ‘Prime Giant’ sweet cherry trees need 780 GDD to complete fruit development, which is easily satisfied in warm climates.

The application of postharvest deficit irrigation with water savings of up to 40% over the control treatment had no significant effect on the carbohydrate content of roots, or on flowering and fruit set. However, it reduced vegetative growth, which decreases the LA:F ratio and could negatively affect fruit size. In order to avoid quality losses, such as small cherries, fruit thinning should be considered in high cropping years in order to achieve LA:F values higher than 180 cm^2^ fruit^−1^, which would ensure a cherry unitary mass above 10 g. High cropping years were the result of a high fruit setting, which was linked to a high concentration of starch and soluble carbohydrates in the roots of the tree. SSCC values of more than 20% in roots led to 33% higher fruit setting values than those measured in years with SSCC values lower than 20%. Double fruit occurrence was not influenced by the irrigation strategies assayed but by a maximum average air temperature, in excess of 30 °C, during the first 30 days after harvest.

## Figures and Tables

**Table 1:** Chilling requirements measured for the November–March period at Jumilla (Spain) and growing degree hours (GDH) between the end of dormancy and full bloom of ‘Prime Giant’ sweet cherry trees.

	Chill Hours	Chill Units	Chill Portions	GDH	Blooming Date
2015	1299	1123	94	6853	April 6th
2016	1181	1038	93	7014	April 16th
2017	1326	1269	96	6363	April 3rd
2018	1274	1087	92	6708	April 12th
Mean	1270	1129	94	6735	
SE	31.4	49.6	0.8	139	
CV	0.02	0.04	0.01	0.02	

Standard error (SE), Coefficient of variation (CV).

**Table 2:** Number of spurs per meter, flower buds per spur, flowers per bud and number of fruits per 100 flowers measured in ‘Prime Giant’/SL64 sweet cherry combination at Jumilla (Spain).

		Spurs per Meter	Flower Buds per Spur	Flowers per Bud	N^0^ Fruits per 100 Flowers
Treatment	CTL	15.77	5.17	3.04	21.93
	RDI	17.43	5.18	3.10	22.06
	SDI	18.33	5.39	3.19	24.87
Year	2015	-	4.79	2.92	15.08
	2016	18.09	5.40	3.48	28.53
	2017	14.93	5.16	3.04	19.16
	2018	18.51	5.63	3.00	29.05
ANOVA	Treatment	n.s.	n.s.	n.s.	n.s.
	Year	n.s.	n.s.	n.s.	***
	T × Y	n.s.	n.s.	n.s.	n.s.

In the ANOVA section, *** refers to significant effect at *p* = 0.001 and n.s. to not significant.

**Table 3:** Lengths of sweet cherry development stages as day of the year (DOY), days after full bloom (DAFB), number of days elapsed between stages (Duration) and growing degree days base 4 °C (GDD).

		DOY	DAFB	Duration	GDD
2015	Full Bloom	96	0	8	74
	Fruit Set	104	8	9	98
	Stage I	113	17	10	128
	Stage II	123	27	7	107
	Stage III	130	34	24	370
	Harvest	154	58	58	777
2016	Full Bloom	107	0	7	72
	Fruit Set	114	7	10	89
	Stage I	124	17	13	140
	Stage II	137	30	9	111
	Stage III	146	39	23	392
	Harvest	169	62	62	805
2017	Full Bloom	93	0	8	75
	Fruit Set	95	8	7	94
	Stage I	105	15	14	121
	Stage II	116	29	8	101
	Stage III	126	37	23	352
	Harvest	153	60	60	743
2018	Full Bloom	102	0	8	76
	Fruit Set	110	8	7	89
	Stage I	117	15	12	128
	Stage II	129	27	8	93
	Stage III	137	35	28	405
	Harvest	165	63	63	791
Total duration	Mean	160	61		779
	SE	8.0	2.2		26.6
	CV	0.050	0.036		0.034

Standard error (SE), Coefficient of variation (CV).
